# Structural Implications for Selective Targeting of PARPs

**DOI:** 10.3389/fonc.2013.00301

**Published:** 2013-12-20

**Authors:** Jamin D. Steffen, Jonathan R. Brody, Roger S. Armen, John M. Pascal

**Affiliations:** ^1^Department of Biochemistry and Molecular Biology, Kimmel Cancer Center, Thomas Jefferson University, Philadelphia, PA, USA; ^2^Department of Surgery, Division of Surgical Research, Jefferson Pancreas, Biliary, and Related Cancer Center, Kimmel Cancer Center, Thomas Jefferson University, Philadelphia, PA, USA; ^3^Department of Pharmaceutical Sciences, Kimmel Cancer Center, Thomas Jefferson University, Philadelphia, PA, USA

**Keywords:** PARP, selectivity, structure, inhibitor design

## Abstract

Poly(ADP-ribose) polymerases (PARPs) are a family of enzymes that use NAD^+^ as a substrate to synthesize polymers of ADP-ribose (PAR) as post-translational modifications of proteins. PARPs have important cellular roles that include preserving genomic integrity, telomere maintenance, transcriptional regulation, and cell fate determination. The diverse biological roles of PARPs have made them attractive therapeutic targets, which have fueled the pursuit of small molecule PARP inhibitors. The design of PARP inhibitors has matured over the past several years resulting in several lead candidates in clinical trials. PARP inhibitors are mainly used in clinical trials to treat cancer, particularly as sensitizing agents in combination with traditional chemotherapy to reduce side effects. An exciting aspect of PARP inhibitors is that they are also used to selectivity kill tumors with deficiencies in DNA repair proteins (e.g., BRCA1/2) through an approach termed “synthetic lethality.” In the midst of the tremendous efforts that have brought PARP inhibitors to the forefront of modern chemotherapy, most clinically used PARP inhibitors bind to conserved regions that permits cross-selectivity with other PARPs containing homologous catalytic domains. Thus, the differences between therapeutic effects and adverse effects stemming from pan-PARP inhibition compared to selective inhibition are not well understood. In this review, we discuss current literature that has found ways to gain selectivity for one PARP over another. We furthermore provide insights into targeting other domains that make up PARPs, and how new classes of drugs that target these domains could provide a high degree of selectivity by affecting specific cellular functions. A clear understanding of the inhibition profiles of PARP inhibitors will not only enhance our understanding of the biology of individual PARPs, but may provide improved therapeutic options for patients.

## Introduction

**Figure 1:**
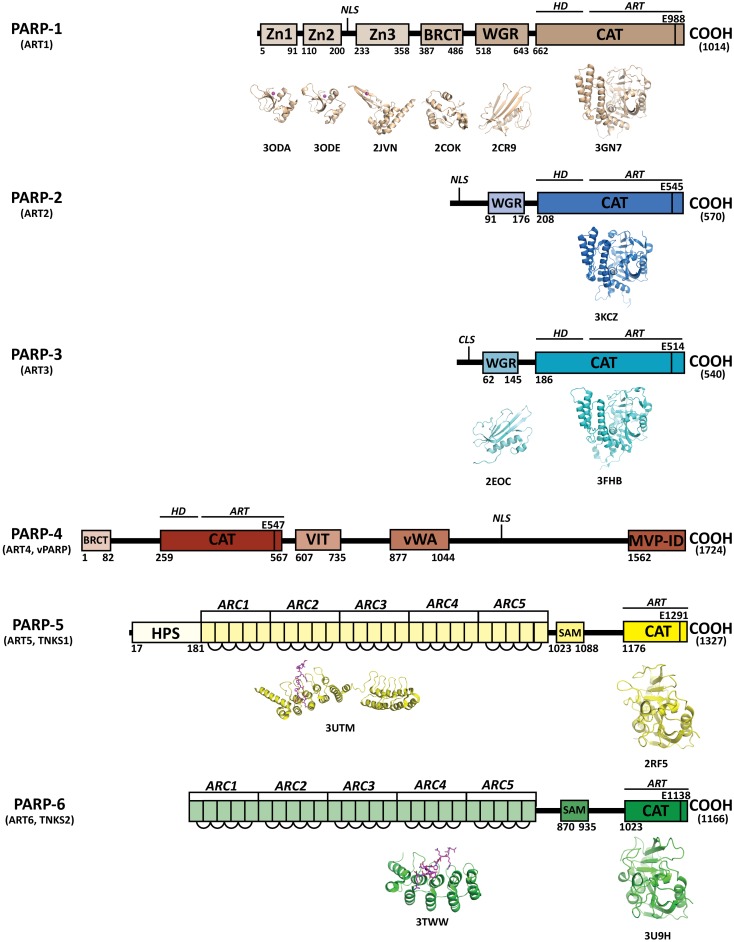
**Domains of human PARPs**. A sequence and structural representation of the six *bona fide* PARPs. Each PARP has a catalytic domain containing an ADP-ribosyltransferase domain (ART) and conserved catalytic glutamic acid residue. In addition PARPs 1–4 contain a helical domain (HD) that serves in allosteric regulation. PARPs 1–3 contain a WGR domain, which is important in DNA-dependent catalytic activation. The breast cancer susceptibility protein-1 C-terminus (BRCT) domain is commonly found in DNA repair and checkpoint proteins, and resides in the automodification domain of PARP-1, and is also present in PARP-4. Zinc-fingers Zn1 and Zn2 of PARP-1 are important in binding DNA, while the third zinc-finger (Zn3) is important in DNA-dependent catalytic activation. Other domains and sequences represented include: centriole-localization signal (CLS), vault protein inter-alpha-trypsin (VIT), von Willebrand type A (vWA), major vault particle interaction domain (MVP-ID), His-Pro-Ser region (HPS), ankyrin repeat clusters (ARCs), sterile alpha motif (SAM), and nuclear localization signal (NLS).

ADP-ribosyltransferases (ARTs) comprise a family of structurally conserved enzymes that catalytically cleave NAD^+^ and transfer the ADP-ribose moiety to acceptor residues of target proteins (1). Poly(ADP-ribosyl) polymerases (PARPs) are a subset of the ART family that continue this reaction to create long chains of linear and/or branched poly(ADP-ribose) (PAR). Currently, only the first six members of this family (ARTs 1–6) are regarded as having poly(ADP-ribosyl)ation activity: PARP-1, PARP-2, PARP-3, PARP-4 (vPARP), PARP-5a (TNKS1), and PARP-5b (TNKS2) (Figure 1). The remaining ARTs 7–17, although originally considered PARPs (PARPs 6–16) (2), are only capable of producing mono-ADP-ribose modifications and are referred to as mono-ARTs (MARTs). ARTs 9 (PARP-9; BAL-1) and 13 (PARP-13) have yet to confirm any sort of catalytic activity like PARPs or MARTs. The degree of ADP-ribosylation in cells is not only controlled by ARTs, but also by PARG and ADP-ribosyl hydrolases that reverse this modification [recently reviewed in Ref. (3)].

Poly(ADP-ribose) polymerase-1 has emerged as a prominent target in chemotherapy due to its important role in maintenance of genomic integrity. Its functional roles in the DNA damage response and cell fate determination have fueled development of PARP-1 inhibitors. Some of these compounds have entered clinical trials with promising therapeutic applications toward treatment of cancer. In combination with DNA damaging agents (e.g., temozolomide, cisplatin) or irradiation, PARP-1 inhibitors are effective chemosensitizers (4). As monotherapy, PARP-1 inhibitors selectively kill tumors harboring DNA repair deficiencies such as genetic deletion of genes involved in the BRCA1 and BRCA2 homologous recombination DNA repair pathway (5, 6). This phenomenon referred to as “synthetic lethality” has attracted clinical attention and has paved the way for a “personalized” approach to cancer therapy (7).

Originally PARP-1 was the only known enzyme with poly(ADP-ribosylation) activity, but as other PARPs began to emerge the selectivity of PARP-1 inhibitors were called into question and now they are typically referred to as PARP inhibitors. In fact, 185 PARP inhibitors were recently evaluated for binding to the catalytic domain of several different PARPs, and revealed binding profiles demonstrating a lack of specificity for any given PARP (8). Where PARPs 1–3 seem to have an important role in maintaining genomic integrity, other PARPs have roles such as telomere replication and cellular transport (9, 10). With such a large family of enzymes carrying out distinct biological functions, drug targeting of the conserved catalytic site of PARPs has raised questions concerning intended pharmacological outcomes. This has led some groups to pursue development of PARP inhibitors with increased selectivity to better understand the biology of targeting individual PARPs.

The aim of this review is to describe the structural relationships among PARPs and the drug design efforts that have found ways to engineer PARP selectivity. We bring attention to non-catalytic domains that are contained within PARPs, and how targeting these domains could provide increased selectivity. The differences in therapeutic benefit and unwanted side effects of selective PARP inhibition versus pan-PARP inhibition is not well understood, and the development and use of more selective agents will ultimately help answer these important questions concerning PARP inhibitors as chemotherapy. For clarity and relevance purposes, all structural comparisons regarding residues and numbering are described based on human PARP-1 unless otherwise noted. The locations of key binding or catalytic site residues have been given position numbers in the text and figures to help guide the viewer through the structural comparisons.

## Structural Similarities and Differences among PARPs

Poly(ADP-ribose) polymerases are multi-domain proteins that are related through their highly conserved ART domain (Figure 1). Outside of the ART domain, distinct domain architectures quickly differentiate the structure and function of each PARP. The catalytic domain crystal structures have been solved for all current PARPs except for PARP-4 (vPARP). The crystal structures of some non-catalytic domains of PARPs have been solved, although there is no crystallographic data on any full-length PARP. The closest to a full-length structure is a catalytically active complex of PARP-1 essential domains bound to DNA damage (11).

### Catalytic domain

**Figure 2:**
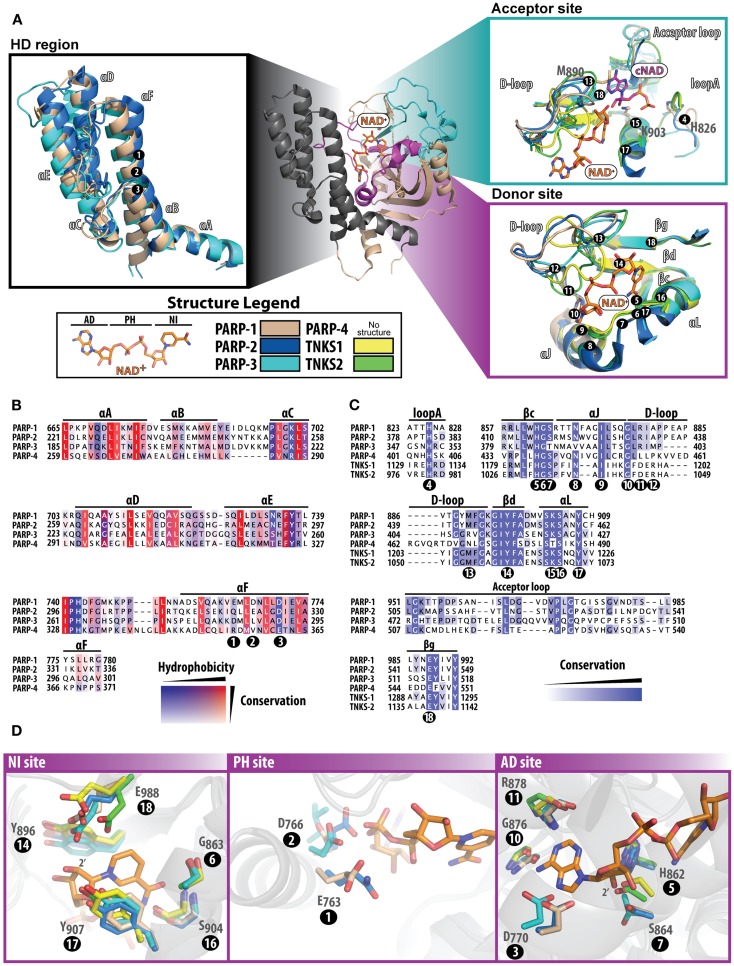
**Structure and sequence comparisons of the PARP catalytic domain**. The PARP-1 catalytic domain [**(A)**; center] is used as a template to compare specific regions among other PARPs. All other PARPs were structurally aligned using Pymol (www.pymol.org/). In **(A)**, all numbering positions corresponding to the protein sequence are labeled at the Cα of the residue in PARP-1. The helical domain [**(A)**; left] present in PARPs 1–4, consists of six alpha helices numbered A–F. At the core of this domain are several hydrophobic residues, which are highly conserved among PARPs **(B)**. The acceptor and donor sites [**(A)**; right] display binding of NAD^+^ (modeled) and the ADP portion of co-crystallized carba-NAD (cNAD) (PDB ID: 1A26). The donor site that binds NAD^+^ is highly conserved **(C)** among all PARPs, although the acceptor site is much less conserved (acceptor loop and loop A). The D-loop assumes varying structural conformations and is also less conserved, which is an indication of where selectivity may be best achieved. The donor site is composed of three regions that bind to NAD^+^ **(D)** the NI site (left), the PH site (middle), and the AD site (right). Multiple sequence alignments were carried out using ClustalW2 [(83); www.ebi.ac.uk/Tools/msa/clustalw2/], and the sequences of human PARPs were analyzed using Jalview [(84); www.jalview.org/]. Structures used for comparisons include: PARP-1 (PDB ID: 3GN7), PARP-2 (PDB ID: 3KCZ), PARP-3 (PDB ID: 3FHB), TNKS1 (PDB ID: 2RF5), and TNKS2 (PDB ID: 3U9H).

While the pairwise sequence identity among the catalytic domains of human PARPs is under 50%, their structures are highly conserved (Figure 1). The PARP catalytic domain contains an ART domain composed of a donor site with a β-α-loop-β-α signature motif that binds NAD^+^, an acceptor site where ADP-ribose chains are extended, and a helical domain (HD) present in PARPs 1–4 and some MARTs (Figure 2A). Although there is no crystal structure of NAD^+^ bound to a human PARP, the diphtheria toxin structure (PDB: 1TOX) of NAD^+^ bound to a bacterial ART domain (12) along with homology modeling of PARP-1 (13) provides insight into the likely binding mode. Within the donor site is a nicotinamide-binding pocket and an ADP-ribose binding pocket. PARPs share an H-Y-E triad sequence motif in their active site that is altered in MARTs. These residues along with other residues conserved among PARPs are critical for the initiation, elongation, and in some instances branching of PAR synthesis (14). Substrate binding in the acceptor site is also not completely understood, since the only structural data shows a portion of a bound non-hydrolyzable NAD^+^ analog (carba-NAD, cNAD) that provides insights into how PAR might bind (15).

### Helical domain

The HD consists of six α-helices (A through F) that form a hydrophobic core, with helix αA contributing to the fold of the ART domain (Figure 2A, HD region). The HD structures of PARP-2 and PARP-3 superimpose with PARP-1 very well, and overall have a high sequence similarity (Figure 2B). In PARPs 1–3 (and likely PARP-4) helix αF is adjacent to the donor NAD^+^ binding site. In PARP-1, structural rearrangement of the N-terminal Zn1, Zn3, and WGR domains in response to DNA damage detection causes a destabilization of the HD that ultimately triggers catalytic hyper-activation (11, 16). While PARP-4 has a putative HD based on sequence alignment, tankyrases do not contain a HD. Outside of PARP-1 DNA-dependent activation, other mechanisms that could destabilize the HD remain unknown. DNA-independent PARP-1 activation from phosphorylation has been reported (17), but the mechanisms that trigger catalytic activation are unclear.

### ART domain – donor site

In the PARP catalyzed reaction, the co-substrate NAD^+^ binds to the ART domain and “donates” the ADP-ribose portion to an amino acid residue or a growing PAR chain (Figure 2A, donor site). The donor site is also the site where PARP inhibitors bind. The donor site is composed of a nicotinamide-binding pocket (NI site), a phosphate binding site (PH site), and an adenine-ribose binding site (AD site) (Figure 2D). The NI site consists of a structural motif that is highly conserved among PARPs: two tyrosine residues that form a π–π stacking interaction with the nicotinamide ring (Figure 2D, positions 14 and 17), and a hydrogen-bond network between a serine hydroxyl (position 16) and glycine backbone atoms (position 6) with the carboxamide of NAD^+^. In the AD site of PARP-1 (Figure 2D), main-chain atoms of Gly876 (position 10) and Arg878 (position 11), and side-chains of Asp770 (position 3), His862 (position 5), and Ser864 (position 7) are predicted to interact with the adenosine portion of NAD^+^. In the PH site (Figure 2D), Asp766 (position 2) and Glu763 (position 1) are situated near the pyrophosphate group of NAD^+^. Based on modeling predictions, the catalytic conserved residues (H-Y-E motif) residing at the NI site include Glu988 (position 18) that binds to the 2′-hydroxyl group of the nicotinamide ribose positioning NAD^+^ for nucleophilic attack by the acceptor substrate (Figure 2D, NI site), His862 (position 5) that binds to the 2′ adenine-ribose hydroxyl (Figure 2D, AD site), and Tyr896 (position 14) that stacks with the nicotinamide ring (Figure 2D, NI site). Similarly, the rest of the donor site is very much the same among PARPs 1–3 with a few minor variations (Figures 2B,C): (i) in the NI site Ser864 (position 7) is replaced with Thr386 (PARP-3), (ii) in the PH site Glu763 (position 1) is replaced with Gln319 (PARP-2), Asp284 (PARP-3), and Arg354 (PARP-4), and (iii) Asp766 in PARP-1 (position 2) extends to Glu322 (PARP-2), Leu287 (PARP-3), and Val357 (PARP-4). Other observations near the donor site that could influence drug selectivity include variations in PARP-3 with respect to PARP-1, such as Val390/Asn868 (position 8) and Met402/Ala880 (position 12).

Like PARPs 1–3, tankyrases contain an ART domain with the catalytic signature (H-Y-E) motif including the active glutamic acid residue essential for PAR synthesis. The NI site is very similar, however since tankyrases do not have an HD domain to form the outer wall of the AD and PH site, residues vary greatly in these regions. Instead, the donor site loop (D-loop, Figure 2A) of tankyrases helps form this outer wall creating a more restricted environment in its closed conformation. Perhaps the most interesting feature of the tankyrase catalytic domains is that they contain a CHCC-type zinc-finger that is not known to be present in any other ART domain (18). The importance of this motif is only speculative, but could be used for structural stability or mediating protein or DNA interactions. The sequence identity between TNKS1 and TNKS2 are highly conserved, with variable residues located mostly outside of the NAD^+^ binding site.

### ART domain – acceptor site

Despite the lack of structural data on substrates bound to the acceptor site of PARPs, a structure has been reported for a transition state analog of NAD^+^ bound to the acceptor site of chicken PARP-1 (15). From this structure of bound cNAD (Figure 2A, Acceptor site), it can be projected that His826 (position 4), Lys903 (position 15) and the backbone amides of 985 and 986 form a H-bond network with the acceptor PAR pyrophosphates. The ribose hydroxyl groups H-bond to Tyr907 (position 17) and Glu988 (position 18), and the adenine base stacks against Met890 (position 13). These residues are conserved in other PARPs with the exception of PARP-3 that does not contain the Met890, which is replaced with an arginine (408) that forms a salt bridge with Asp455 (19). This amino acid change could contribute to the smaller polymers produced by PARP-3 (20, 21). A highly variable region among PARPs is in the acceptor loop (Figure 2A, acceptor site and Figure 2C). PARP-2 has a similar alignment as PARP-1 but contains an additional three residues in this loop, most notably an additional tyrosine residue (Tyr539) that projects into the acceptor site based on the structure of mouse PARP-2 (22). Both tankyrases have a much shorter acceptor loop and diverge in their structural alignment with PARP-1. These differences in the acceptor loop across PARPs could potentially specify a preference for particular proteins that are targeted for modification.

### ART domain – D-loop

The D-loop lines the donor site and partially the acceptor site, and represents structural diversity among PARPs due to variations in conformations observed across structures (Figure 2A). The D-loop in PARPs 1 and 2 are near identical; in contrast, the D-loop of PARP-3 (Gly398-Lys411) is smaller than PARP-1, which leaves the donor site more open (19). The major differences comparing PARP-3 to PARP-1 include the Met402/Ala880 (position 12) and Gly406/Tyr889 changes. The D-loop of tankyrases is frequently observed in a closed conformation, which blocks the NAD^+^ binding site, although it is likely that this loop is dynamic to allow NAD^+^ access (18). The sequence conservation between tankyrases is very similar, although in structures of TNKS1 the D-loop is positioned closer to the nicotinamide-binding pocket and in TNKS2 it closes near the ADP-ribose binding pocket. The differences between TNKS1 and TNKS2 may reflect an inherent mobility of the tankyrase D-loops.

### Non-catalytic domains

Poly(ADP-ribose) polymerase-1 is the founding member and most studied of the PARP family. PARP-1 and PARP-3 are the only PARPs for which structures of all domains are known (Figure 1). PARP-1 has a modular domain architecture comprising five domains in addition to the catalytic domain: N-terminal Zn1 and Zn2 domains which are homologous zinc-finger domains that recognize damaged DNA ends (23), a third zinc-finger domain (Zn3) that is important in DNA-dependent activation (24), a central BRCA C-terminus-like fold (BRCT) domain that mediates protein–protein interactions and serves as a substrate for PAR automodification (25), and a tryptophan-glycine-arginine (WGR) domain that interacts with DNA and is important for DNA-dependent activation.

As in PARP-1, both PARP-2 and PARP-3 share a homologous WGR domain positioned N-terminal to the catalytic domain. In PARP-1 the WGR domain is important for DNA-dependent activation and interacts with DNA (11). The function of WGR in PARP-2 and PARP-3 is not well evaluated, although it likely interacts with DNA based on homology to PARP-1. Neither PARP-2 or PARP-3 have zinc-finger binding domains or a BRCT domain, but PARP-2 has a highly basic N-terminal region that could mediate interaction with DNA.

Originally characterized by its association with major vault protein (MVP) through its MVP interaction domain (MVP-ID) (26), the structure and function of PARP-4 is one of the least understood of the PARPs. Other PARP-4 domains include vault protein inter-alpha-trypsin (VIT) and von Willebrand type A (vWA) domains that are also found together in the inter-alpha-trypsin inhibitor (ITI) family, but are not completely understood in connection with PARP-4. It is not known to contain zinc-fingers or a WGR domain, but contains an N-terminal BRCT domain homologous to PARP-1 (26).

While tankyrases contain a catalytic domain that is capable of producing PAR, they do not share any other domains with the other PARPs. With regard to the PARPs, tankyrases have the following unique domains: an ankyrin repeat region that binds acceptor proteins, a sterile alpha motif (SAM) domain that mediates oligomerization, and a histidine-proline-serine rich (HPS) domain unique to TNKS1 with unknown function (9). The series of ankyrin repeats are arranged into five ankyrin repeat clusters (ARCs). With the exception of ARC3, each ARC is reported to bind acceptor proteins that carry the tankyrase consensus binding sequence RXXPDG (27). The tankyrase targets, Axin1 and peptides derived from several other target proteins, have been co-crystallized with individual ARCs, and the structures illustrate the key features of the binding interaction (28, 29). The overall conformation of the five ARCs and possible structural arrangements upon mediating protein–protein interactions is not currently understood. TNKS2 is nearly identical to TNKS1 except that it does not have an HPS region and has a seven amino acid insertion after the ankyrin repeat region with unknown importance (30).

## Development of Selective PARP Inhibitors

**Figure 3:**
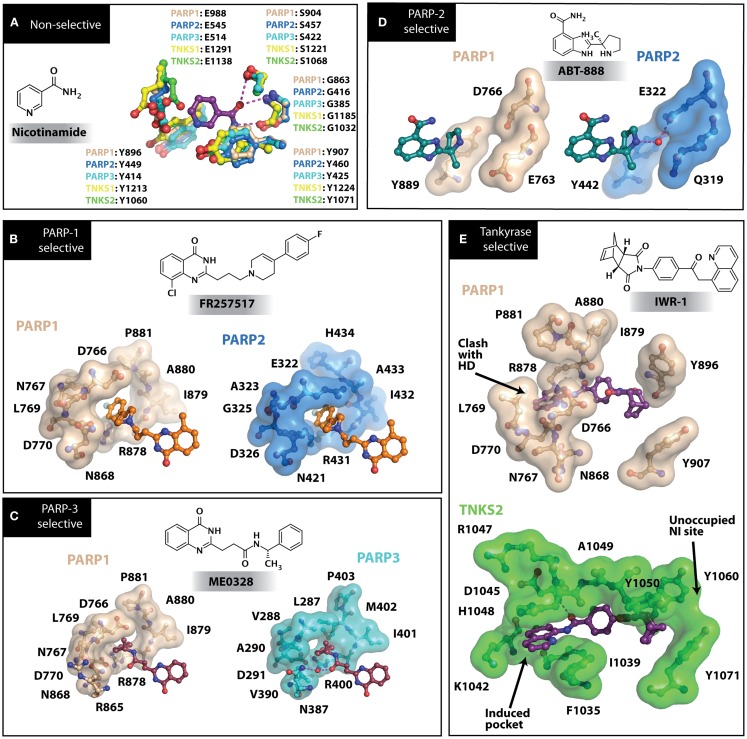
**PARP inhibitors bound to the catalytic domain of PARPs**. Non-selective inhibitors such as nicotinamide [**(A)**, non-selective] only interact with the nicotinamide pocket (NI site), which is a highly conserved region. Most developed PARP inhibitors have been designed to bind the NI site and adjacent sites to gain potency and selectivity. The compound FR257517 contains a fluorophenyl that reaches into the ADP-ribose binding site (AD site) of PARP-1 (PDB ID: 1UK0) to gain selectivity [**(B)**, PARP-1 selective]. An aligned PARP-2 structure (PDB ID: 3KCZ) shows how the AD site is very similar to that of PARP-1, but the increased hydrophobicity of the PARP-1 AD site is attributed to the observed PARP-1 selectivity. Compounds that interact with E322 of PARP-2 (PDB ID: 3KJD) can gain selectivity over PARP-1 due to the differences in distance between this acidic side-chain and drug heteroatoms [**(D)**, PARP-2 selective]. PARP-3 (PDB ID: 4GV4) has a structurally similar AD site as PARPs 1 and 2, although residue variation creates an environment distinct in polarity that guides selectivity [**(C)**, PARP-3 selective]. Tankyrase inhibitors often demonstrate a much higher window of selectivity from PARPs 1–4, although selectivity between TNKS1 and TNKS2 is difficult to obtain. IWR-1 is a non-traditional PARP inhibitor in that it does not target the nicotinamide site of TNKS2 [**(E)**, Tankyrase selective]. PARP-1 (PDB ID: 1UK0) was aligned with the co-crystallized TNKS2 structure containing IWR-1 (3UA9) to demonstrate that the quinoline ring clashes into the AD site of PARP-1 due to the presence of its helical domain.

Nearly 30 years ago inhibitors of PARP-1 were discovered, and shown to sensitize cells to DNA damaging agents (31). These early PARP inhibitors, such as the benzamides and isoquinolinones, established a core pharmacophore from which future PARP inhibitors would build (32, 33). Co-crystallization of the catalytic domain of chicken PARP-1 with these inhibitors showed anchoring into the nicotinamide-binding pocket of PARP-1, consistent with the nicotinamide-mimicking pharmacophore (13, 34). The carboxamide functional group of nicotinamide makes three hydrogen-bond interactions with the serine hydroxyl and glycine backbone atoms of the NI site, and the benzene ring makes π–π stacking interactions with surrounding tyrosine residues (Figure 3A). The chemotherapeutic potential of PARP inhibitors prompted medicinal chemistry efforts aimed at designing newer PARP inhibitors with improved potency and pharmacokinetic properties. These efforts spurred development of several small molecule nicotinamide-like scaffolds with modified side groups reaching outside of the pocket and into regions such as the donor AD site that improved potency, selectivity, and bioavailability (Table 1). For more information on this development the reader is referred to an in-depth review focusing on the optimization of PARP inhibitors (35).

**Table 1:** **Selectivity of PARP inhibitors. Published IC50 values of PARP inhibitors that have been tested against multiple PARPs**.

PARP-1 SELECTIVE										
Compound	Structure	Class	PARP-1	PARP-2	PARP-3	PARP-4	TNKS1	TNKS2	Selectivity^a^	Reference
		
			IC50 (nM)					
DR2313	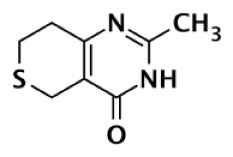	Thiopyranopyrimidine	200	2,400					12	Nakajima et al. (81)
1	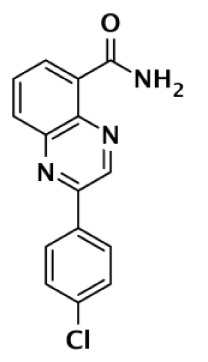	Quinoxaline	30	90					3	Sunderland et al. (85)
FR257517	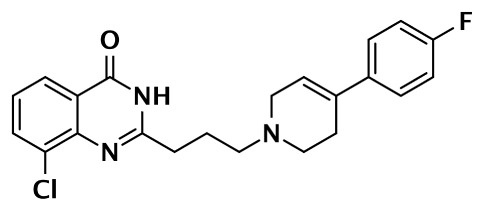	Quinazolinone	13	500					39	Ishida et al. (40)
BYK204165	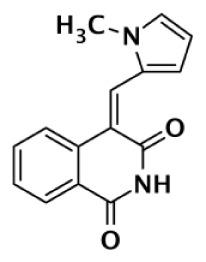	Isoquinolindione	45	4,000					89	Eltze et al. (41)
BYK49187	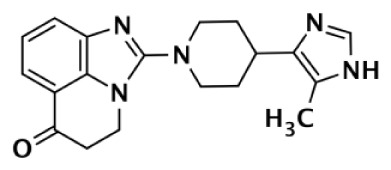	Imidazoquinolinone	4	20					5	Eltze et al. (41)
BYK20370	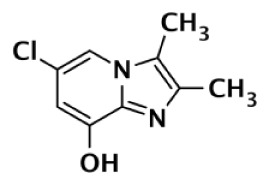	Imidazopyridine	400	2,000					5	Eltze et al. (41)

**PARP-2 SELECTIVE**										
**Compound**	**Structure**	**Class**	**PARP-1**	**PARP-2**	**PARP-3**	**PARP-4**	**TNKS1**	**TNKS2**	**Selectivity^b^**	**Reference**
		
			**IC50 (nM)**					

Olaparib (AZD-2281) (KU-0059436)	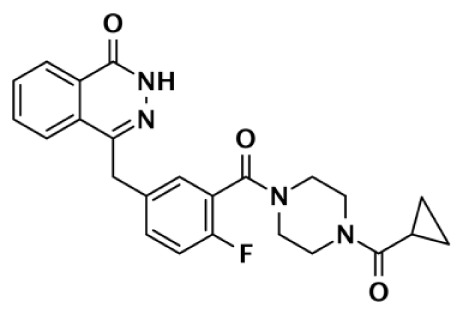	Pthalazinone	5	1			1,500		5	Menear et al. (86)
Veliparib (ABT-888)	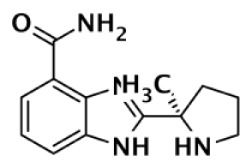	Benzimidazole	5	2					2.5	Penning et al. (87)
			8.3	11			14,970	6,519	0.75	Huang et al. (43)
2	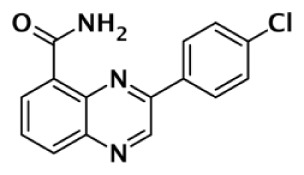	Quinoxaline	101	8					13	Ishida et al. (40)
3	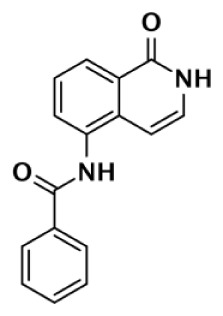	Isoquinolinone	13,900	1,500					9	Sunderland et al. (85)
4	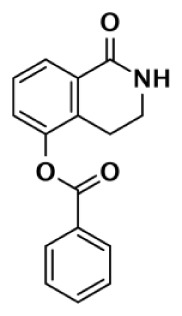	Isoquinolinone	13,000	800					16	Pellicciari et al. (44)
5	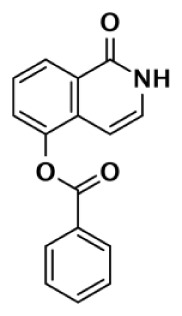	Isoquinolinone	9,000	150					60	Pellicciari et al. (44)

**PARP-3 SELECTIVE**										
**Compound**	**Structure**	**Class**	**PARP-1**	**PARP-2**	**PARP-3**	**PARP-4**	**TNKS1**	**TNKS2**	**Selectivity^c^**	**Reference**
		
			**IC50 (nM)**					

ME0328	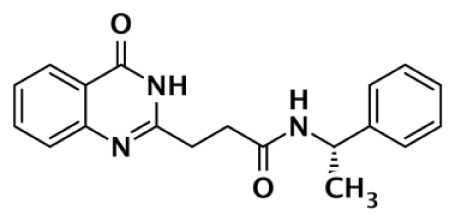	Quinazolinone	6,300	10,800	890	>30,000	>30,000	>30,000	7	Lindgren et al. (45)

**PARP-1 AND PARP-2 SELECTIVE**										
**Compound**	**Structure**	**Class**	**PARP-1**	**PARP-2**	**PARP-3**	**PARP-4**	**TNKS1**	**TNKS2**	**Selectivity^d^**	**Reference**
		
			**IC50 (nM)**					

GPI6150	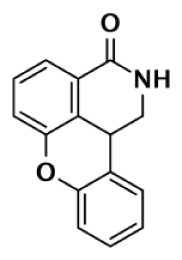	Isoquinolinone	∼100	∼100					n.d.^f^	Zhang et al. (82)
Niraparib (MK-4827)	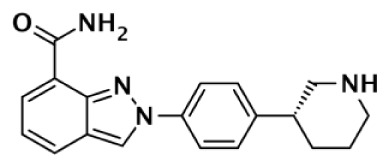	Indazole	3.8	2.1	1,300	330	570		87–342	Jones et al. (88)
5-AIQ	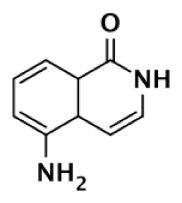	Isoquinolinone	940	1,050					n.d.^f^	Sunderland et al. (85)
PJ-34	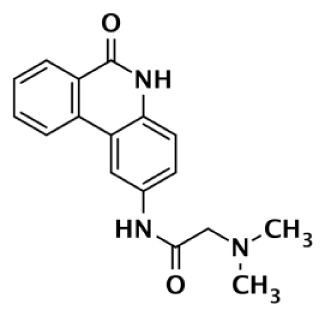	Phenanthridine	600	1,000					n.d.^f^	Pellicciari et al. (44)
DPQ	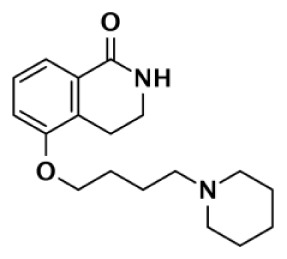	Isoquinolinone	4,500	5,300					n.d.^f^	Pellicciari et al. (44)
6	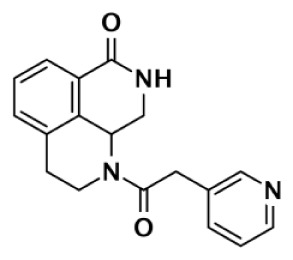	Benzo-naphthyridinone	1	1	50	440	3,500		50–3,500	Torrisi et al. (89)

**TANKYRASE SELECTIVE**										
**Compound**	**Structure**	**Class**	**PARP-1**	**PARP-2**	**PARP-3**	**PARP-4**	**TNKS1**	**TNKS2**	**Selectivity^e^**	**Reference**
		
			**IC50 (nM)**					

XAV939	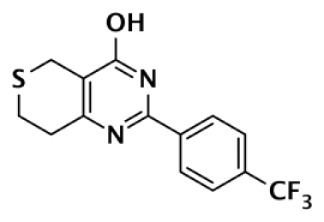	Pyrimidinone	2,194	114			11	4	200	Huang et al. (43)
			620				14	8	44	Karlberg et al. (53)
			120	46	>10,000		11	8	11	Larsson et al. (90)
7	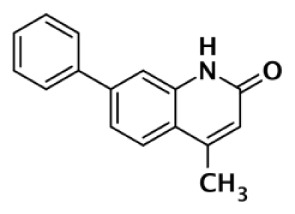	Isoquinolinone	>10,000	>10,000	>10,000		860	52	12	Larsson et al. (90)
IWR-1	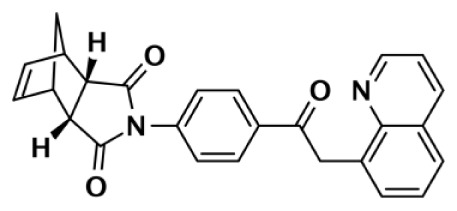	Tetrahydro-Phthalimide	>18,750	>18,750			131	56	>143	Huang et al. (43)
			>85,000	>170,000			150	39	>567	Bregman et al. (54, 55)
8	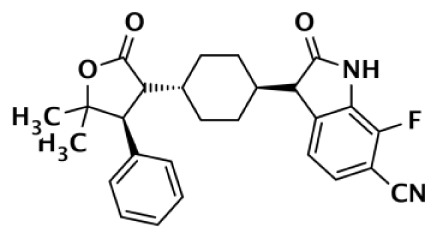	Oxazolidinone	>85,000	>170,000			1		>85,000	Bregman et al. (54, 55)
9	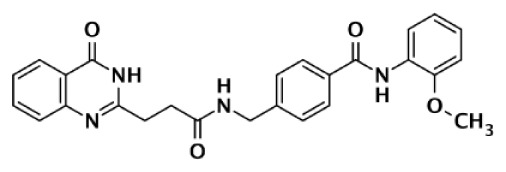	Quinazolinone		931			8	2	116	Bregman et al. (54, 55)

*^a^ Fold selectivity for PARP-1 vs. PARP-2 (PARP-2 IC50 / PARP-1 IC50)*.

*^b^ Fold selectivity for PARP-2 vs. PARP-1 (PARP-1 IC50 / PARP-2 IC50)*.

*^c^ Fold selectivity for PARP-3 vs. PARP-1 (PARP-1 IC50 / PARP-3 IC50)*.

*^d^ Fold selectivity for PARP-1 vs. PARP-3, PARP-4, and TNKS1 (PARP-1 IC50 / PARP-3, PARP-4, or TNKS1 IC50)*.

*^e^ Fold selectivity for TNKS1 vs. PARP-1 (PARP-1 IC50 / TNKS1 IC50)*.

*^f^ Not determined*.

### Selectivity between PARP-1 and other PARPs

By the late 90s, the identification of a second PARP, termed PARP-2, was reported (36). Since PARP-2 carries out the same catalysis as PARP-1, uses the same co-substrate, and is highly homologous, it is not surprising that most PARP inhibitors show similar inhibition potency between both PARPs. The nicotinamide pockets of PARP-1 and PARP-2 are nearly identical, and there are only minor differences in their ADP-ribose binding pockets. The minor sequence variation, Glu763/Gln319 in HD helix αF, and the presence of Tyr539 of PARP-2 in the acceptor loop, have been noted as important differences in which selectivity could be achieved (22).

Soon after the discovery of PARP-2, several nicotinamide-mimicking inhibitors discovered through a high-throughput cell-based assay identified that most had similar inhibition between PARP-1 and PARP-2, although minor selectivity was noted with certain compounds (37). These findings demonstrated that PARP selectivity could be achieved despite nearly identical binding sites. Although infrequently reported, most compounds that inhibit PARP-1 have little to no preference for PARP-1 over PARP-2. Attempts to improve selectivity resulted in nicotinamide-based compounds that also target outside of the NI site. The quinazolinone-based inhibitor (FR257517) binds the PARP-1 nicotinamide pocket and further interacts with Asn767, Asp770, Asp766, Asn868, and Ala880 in the AD site through its extended substitution (38) (Figure 3B). Interestingly, the extended portion of the molecule induces a conformational change in Arg878 that opens a new hydrophobic pocket surrounded by residues Leu769, Ile879, and Pro881 (Figure 3B). It is thought that a Leu769/Gly325 variation in the induced hydrophobic pocket creates a more hydrophobic environment in PARP-1, which is why this compound is 10-fold more selective for PARP-1 (39, 40). Further modifications of this compound near the NI site accomplished selectivity for PARP-1 up to 39-fold, indicating that selectivity may also be adjusted through modifications near the nicotinamide pocket. Another example is an isoquinolindione compound (BYK204165) that was identified with a 100-fold PARP-1/PARP-2 selectivity (41). Unfortunately there is no co-crystal structure data of this compound to understand this preference.

Most inhibitors developed target PARP-1 and PARP-2 closely, but there are also varying degrees of selectivity for the other PARPs due to the similarities in active sites (although much less frequently reported). Small, basic PARP inhibitors that target the nicotinamide site (such as 3-amino-benzamide) are very unselective across PARPs, and even MARTs. Potent PARP-1 inhibitors with bulky side groups or extensions typically gain selectivity against other PARPs (especially the tankyrases) due to steric clash that can be easily rationalized considering the noticeable structural differences outside of the NI site (Figure 2A).

### Specific PARP inhibitors

Poly(ADP-ribose) polymerases-2 selective inhibition was seen early on with quinoxaline based inhibitors (39). Preference for PARP-2 over PARP-1 is seen based on residue variations between the two. The modified quinoxaline phenyl ring of compound 2 (Table 1) more favorably interacts with the space between Gln319 and Glu322 in PARP-2 over the Glu763 and Asp766 in PARP-1 (as seen in Figure 3D for ABT-888). Also, PARP-2 forms a water-mediated hydrogen-bond with the inhibitor through its acidic residue Glu322, which is not formed by PARP-1, thus creating a stronger affinity for PARP-2. In PARP-1 this residue is a shorter Asp766 residue that is further from the NI site, which may explain the preference for PARP-2 selectivity through a closer, thus stronger interaction (40). Crystallographic studies of ABT-888 also suggest a closer proximity of Glu335 over Asp766 in PARP-1 to the side group N-heteroatom of ABT-888 setting up a potentially more favorable interaction (Figure 3D) (42). Interaction with this acidic residue is essential for potency in many compounds, and may in part explain the near 1000-fold higher selectivity of ABT-888 for PARP-1 and PARP-2 over TNKS1 and TNKS2, which do not have this residue (43).

A library of isoquinolinone derivatives was reported to display selectivity for PARP-2 up to 60-fold (44). This discrimination is thought to be due to a single residue variation of Glu763 in PARP-1 to Gln319 in PARP-2. Interestingly, desaturation of the nicotinamide-mimicking portion also increased PARP-2 selectivity, indicating that even though these sites are highly conserved, small steric effects can have a significant impact on selectivity (44).

Although there is a lack of data on PARP-3 inhibition, recently reported quinazoline derivatives, such as ME0328 (Table 1; Figure 3C), have been shown to have up to sevenfold selectivity for PARP-3 over PARP-1 (45). These compounds anchor into the NI site and extend into the AD donor site of PARP-3, which is slightly larger and more hydrophobic. Differences in polarity and geometry of the AD sites of PARP-3 and PARP-1 are likely guiding factors in the observed selective inhibition. Co-crystallization studies of PARP inhibitors with PARP-3 also indicate that the sequence variation and D-loop conformation changes in the AD site create distinguishing environments for designing PARP-3 selective inhibitors (19). Modifications of the core scaffold that reach out into the acceptor site could target Arg408 (which is a methionine residue in other PARPs) in order to achieve selectivity.

Due to the smaller and more hydrophobic donor site of tankyrases, selectivity over other PARPs can be more easily achieved. The first selective tankyrase inhibitor to be discovered was XAV939, which binds the nicotinamide pocket (46) and has a 200-fold selectivity over PARP-1 (43). Therapeutic interest in tankyrases prompted high-throughput screening (HTS) assays leading to the discovery of IWR-1 (47), JW55 (48), and flavones (49) as specific tankyrase inhibitors. IWR-1 and IWR-2 are non-traditional inhibitors that bind to the AD and PH site but not the NI site of tankyrases, but still block NAD^+^ binding (Figure 3E) (50, 51). IWR compounds bind to the donor site of TNKS1 making H-bond interactions with Tyr1213 and Asp1198 (Tyr1060 and Asp1045 in TNKS2), and stacking interactions between Phe1198 and His1201 (Phe1035 and His1048 in TNKS2) (51, 52). In co-crystal structures, rearrangement of the tankyrase D-loop (Ala1202–Ala1210 in TNKS1; Ala1049–Ala1057 in TNKS2) is observed in which Tyr1203 (Tyr1050 in TNKS2) flips outward allowing access to the binding site, and movement of Phe1198 (Phe1035 in TNS2) creates an induced pocket that accommodates binding (18, 51, 52). In the absence of inhibitor or NAD^+^, Tyr1203 lies across the NAD^+^ binding pocket and forms a hydrogen-bond to the main-chain of Tyr1224 (Y1071 in TNKS2), which effectively blocks access of NAD^+^ to the binding pocket. The opening of this site is similar to the effects seen with XAV939 binding to TNKS2 and TNKS1 (46, 53). In PARPs 1–4 the outer wall is formed in part by the HD that would creates steric clash with these compound (as observed with the aligned PARP-1 structure in Figure 3E). While this molecule is a useful tool for selective tankyrase inhibition, it suffers from poor cellular potency and efforts are being made to improve its potency and pharmacokinetic and pharmacodynamic properties (compounds 8 and 9 from Table 1) (54, 55).

### Other PARP inhibitors that do not mimic nicotinamide

While tankyrase inhibitors appear to be paving the way for non-nicotinamide-based PARP inhibitors, we make note of a few other non-traditional scaffolds. For example, imidazoquinolinones and imidazopyridine based compounds do not contain the carboxamide feature, but are potent inhibitors of both PARPs 1 and 2 (41). These compounds inhibit competitively, meaning they block NAD^+^ from binding and thus would likely have similar challenges as most PARP inhibitors in optimizing selectivity.

Metabolites of coumarin derivatives made way for C-nitroso derivatives that irreversibly inhibited PARP-1. These compounds were observed to eject the zinc ion from the first zinc-finger domain (Zn1), presumably through oxidation of the coordinating cysteine residues resulting in disulfide bond formation (56). This mechanism was noted to act selectively on Zn1 and not Zn2, which fell in line with the loss in catalytic activity but remaining DNA binding (57). These compounds showed promising chemotherapeutic potential as they induced apoptosis in human tumor cells (58). Further development of this molecule resulted in 4-iodo-3-nitroso-benzamide (INO_2_BA; iniparib), a clinical candidate that showed clinical benefit in treating metastatic triple negative breast cancer (TNBC) (59); however a larger phase 3 trial failed to reproduce prolonged survival in TNBC. Iniparib was later demonstrated to have poor selectivity and potency for PARP-1 zinc-fingers (60, 61), and thus is not a “*bona fide*” PARP inhibitor. Unfortunately this drug provided an inaccurate representation of true PARP inhibitors to the community, and its failure does not reflect the therapeutic potential of PARP inhibitors.

### Potential for alternative inhibitors as isoform specific PARP inhibitors

High-throughput screening for PARP catalytic site inhibitors and substrate mimicry are two typical strategies taken to develop new PARP inhibitors. When PARP selectivity is desired, chemical manipulations by side group modification or scaffold optimization are used to target the slight differences in the NAD^+^ binding site. With development of new screening assays, we will be capable of searching for compounds that inhibit non-catalytic domains of PARPs. For example, our group has recently developed an HTS assay to detect allosteric regulation of PARP-1 (62). Since the domains involved in allosteric regulation are unique to PARP-1, identified inhibitors would likely be highly selective. In addition to isoform specificity, inhibition of allosteric regulation may only affect certain functions of PARP-1. For instance, we find that inhibition of allosteric regulation affects DNA-dependent activation without affecting androgen receptor-mediated transcriptional activities. It is likely that other PARP-1 mediated functions would also not be affected by disruption in allosteric regulation, which could be beneficial in terms of pharmacological efficacy and adverse effects.

**Figure 4:**
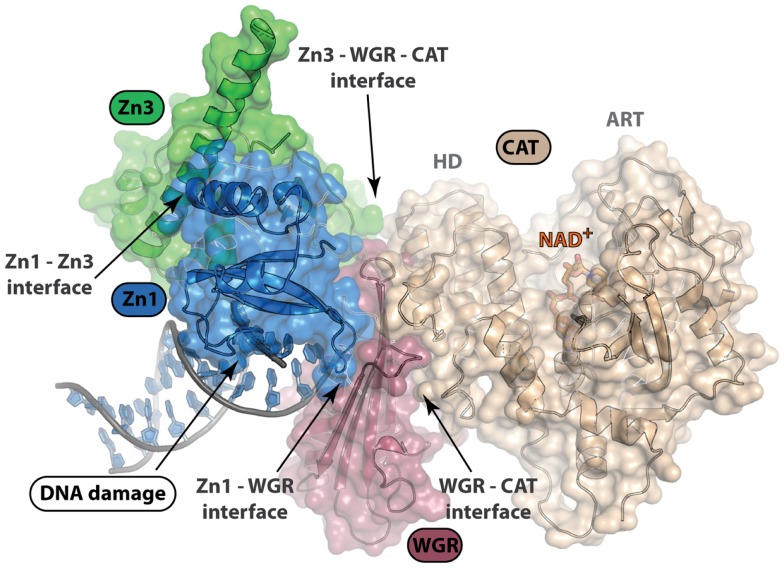
**Structure of PARP-1 in complex with DNA damage**. PARP-1 binds DNA damage and activates catalytic activity nearly 500-fold. Only four of the six domains of PARP-1 (Zn1, Zn3, WGR, and CAT) are essential for DNA-dependent PARP-1 activation. This structure depicts complex formation and protein–protein interactions between domains upon DNA damage recognition (PDB ID: 4DQY). Disruption of these interdomain protein interfaces could be of interest in selective, allosteric targeting of PARP-1. An understanding of the arrangement of PARP-1 domain architecture in the absence of DNA damage recognition will be important for rational drug design efforts targeting protein interactions.

Structural characterization of PARP non-catalytic domains in complex with protein or DNA has provided grounds for rational drug design approaches. Despite difficulties in development of inhibitors that target protein–protein or protein–DNA interfaces, identification of clustered protein interface regions of high-affinity, known as “hot spots,” has been a guiding concept in the inhibition of protein interactions with small molecules (63). From the structure of the essential domains of PARP-1 in complex with DNA damage, there are several domain–domain interfaces that form critical contacts that are required for PARP-1 activation (Figure 4). All-atom molecular modeling analysis of the energetic contribution of individual residues to these protein–protein interfaces predicts that hot spots exist between the domains of PARP-1 (unpublished data). Our analysis using the CHARMM force field and the GBMV implicit solvent model (64, 65) suggested that the majority of binding free energy between the Zn1 and Zn3 domain comes from a few local residues (e.g., R78 and W79 of Zn1). Interestingly, mutation of either of these residues is detrimental to PARP-1 DNA-dependent catalytic activity (62). A small nearby hydrophobic groove exists next to these residues, which could potentially bind and disrupt the interaction between the Zn1 and Zn3 domains (Figure 4). Moving forward, a better understanding of the dynamics of PARP-1 domain arrangements in a cell-based context will be important in any kind of rational drug design approach that targets interdomain interfaces. Furthermore, additional structural studies that can locate the positions of the Zn2 and BRCT domain might also reveal additional domain interfaces.

Another strategy to target PARPs specifically is through the acceptor site in the catalytic domain. This region likely forms contacts with target proteins to be modified with ADP-ribose. The diversity of the region in comparison to the NAD^+^ binding site among PARPs presents a greater potential to achieve selectivity. Unfortunately, the differences in protein target recognition among PARPs are not well understood. It is likely that both sequence and structure play a part in target recognition. We do know that glutamic acid, aspartic acid, and lysine residues are the preferred amino acids that get modified by PARPs (66–70). Small peptides with an ADP-ribose modified glutamic acid or lysine residue could serve as a prototype scaffold for development of such inhibitors.

## Perspective on the Therapeutic Potential of Pan-PARP Inhibitors Versus Selective PARP Inhibitors

Comparisons between the effects of pan-PARP inhibitors and selective PARP inhibitors are largely unknown. In the case of PARP-1, the roles of recognition of DNA damage and repair in the base excision pathway are well established (71). Generation of single-strand breaks (SSBs) tend to accumulate in cells treated with PARP inhibitors, but this is not the case in cells treated with PARP-1 siRNA (72). RNAi technology however, requires careful interpretation since it is a knockdown and not a complete knockout, and even weak PARP-1 activity is enough for efficient DNA repair (73). The residual DNA repair activities of PARP-2 could explain SSB accumulation in cells treated with a PARP inhibitor (that inhibits both PARP-1 and PARP-2), and not in the case of PARP-1 depletion. Another model explains the retention of SSBs by proposing that PARP inhibitors trap PARP-1 and PARP-2 on SSB intermediates and prevent proper repair (72, 74, 75).

In terms of therapeutic potential, PARP inhibitors are more effective at killing BRCA deficient cells than with PARP-1 knockdown (5, 6). A number of clinical trials (Phase I–II) testing PARP inhibitors (with proven activity against either PARP-1 alone or both PARP-1 and 2) singly or in combination with chemotherapy are ongoing (76). Some clinical trials are upfront selecting for patients with known BRCA-deficiency or assessing biomarkers in a retrospective manner; and early reports suggest that selected BRCA-mutant patients do gain the best clinical benefit (77). The selectivity and usefulness of leading clinical PARP inhibitors (veliparib, olaparib, rucaparib) will soon become apparent as clinical trials successfully accrue patients. Moreover, as the research community discovers more BRCA2-related genes (such as the Fanconi Anemia genes) and pathways disrupted in cancers (78) two new opportunities will be: (i) to select patients’ tumors that would be optimal for a synthetic lethal approach using PARP inhibitors and (ii) defining new targets within this pathway (79). Additionally, we are hopeful that with an in-depth understanding of the structure-function of each PARP family member, better and more specific targeting strategies will emerge. Finally, we may be better able to enhance PARP inhibitor-based therapies by taking into account the interplay between the DNA damage response and cell cycle dynamics (e.g., WEE1 inhibitors) (80).

## Conclusion

Over 40 years of research invested from groups worldwide has advanced our understanding of poly(ADP-ribosyl)ation in cancer, identifying PARP-1 as a promising therapeutic target. As the family originating with PARP-1 has grown into a superfamily of PARPs and related MARTs, new therapeutic opportunities have surfaced along with new therapeutic challenges. Since most PARP inhibitors have varying selectivity among PARPs (8), interpretation of biological effects can present difficulties. Only recently have we begun to understand how different PARP inhibitors affect individual PARP function, and whether added therapeutic benefits result from pan-PARP inhibition remains to be determined.

Selectivity of compounds for one PARP over another is infrequently shown, although selectivity between PARP1 and PARP2, and in some instances other PARPs, is becoming more frequently reported. The use of selective agents will be extremely important in understanding each PARPs function. For example, the selectivity of compounds between PARP1, PARP2, and PARP3 is especially needed to clarify roles in response to DNA damage. Methods for screening the family of PARPs has become more prevalent, which will help accelerate the development of selective inhibitors. Cross-inhibition with other enzymes that use NAD^+^ as a substrate or cofactor (such as ADP-ribosylcyclases and sirtuins) is an important concern, but is not typically seen (81, 82).

On the road to PARP selective inhibitors, most efforts will likely continue to focus on modifications of the nicotinamide-based inhibitors. The newer tankyrase selective compounds (such as IWR-1) that target the AD and PH sites but not the NI site present exciting new alternatives to nicotinamide-based inhibitors. It will be interesting to see if similar approaches are effective in other PARPs to promote selectivity. The acceptor sites among PARPs contain varying degrees of differences, which could guide the specificity of modifying target proteins. Targeting features of this region, such as the unique Arg408 residue in PARP-3, could be another way to obtain selectivity. Finally, we bring attention to targeting non-catalytic domains as a route to achieving selectivity. PARPs are the most diverse outside of their catalytic domain, and it is becoming increasingly appreciated that these domains make DNA and protein interactions important for proper function. Targeting non-catalytic domains may even allow us to target specific PARP functions, opening up a new dimension of therapeutic opportunities.

## Conflict of Interest Statement

The authors declare that the research was conducted in the absence of any commercial or financial relationships that could be construed as a potential conflict of interest.
